# Supplementary material for: Increases in the Macrolide Resistance of Mycoplasma genitalium and the Emergence of the A2058T Mutation in the 23S rRNA Gene: Clonal Spread?
Source: Antibiotics (Basel). 2022 Oct 27;11(11):1492. doi: 10.3390/antibiotics11111492 (PMC9686820; doi:10.3390/antibiotics11111492)
Supplement: Supplementary file 1 [file antibiotics-11-01492-s001.zip › antibiotics-1964024-supplementary.pdf]

Supplementary Figure S1. Phylogenetic analysis of the MG191 locus of the adhesion gene (281 bp) in the 27 strains of *Mycoplasma genitalium* with A2058T mutations in the 23S rRNA gene detected in Gipuzkoa (2014-2021). Sequences are represented by the case number. The tree was constructed in BioEdit and MEGA 7 software, using the neighbour-joining method with 1000 bootstrap replications. The column on the right of the tree lists the genotypes identified using the MultAlin and BLAST programs (238-241 being new genotypes not previously described).
* *M. genitalium* G37 (GenBank accession number L43967) was used as the reference strain.

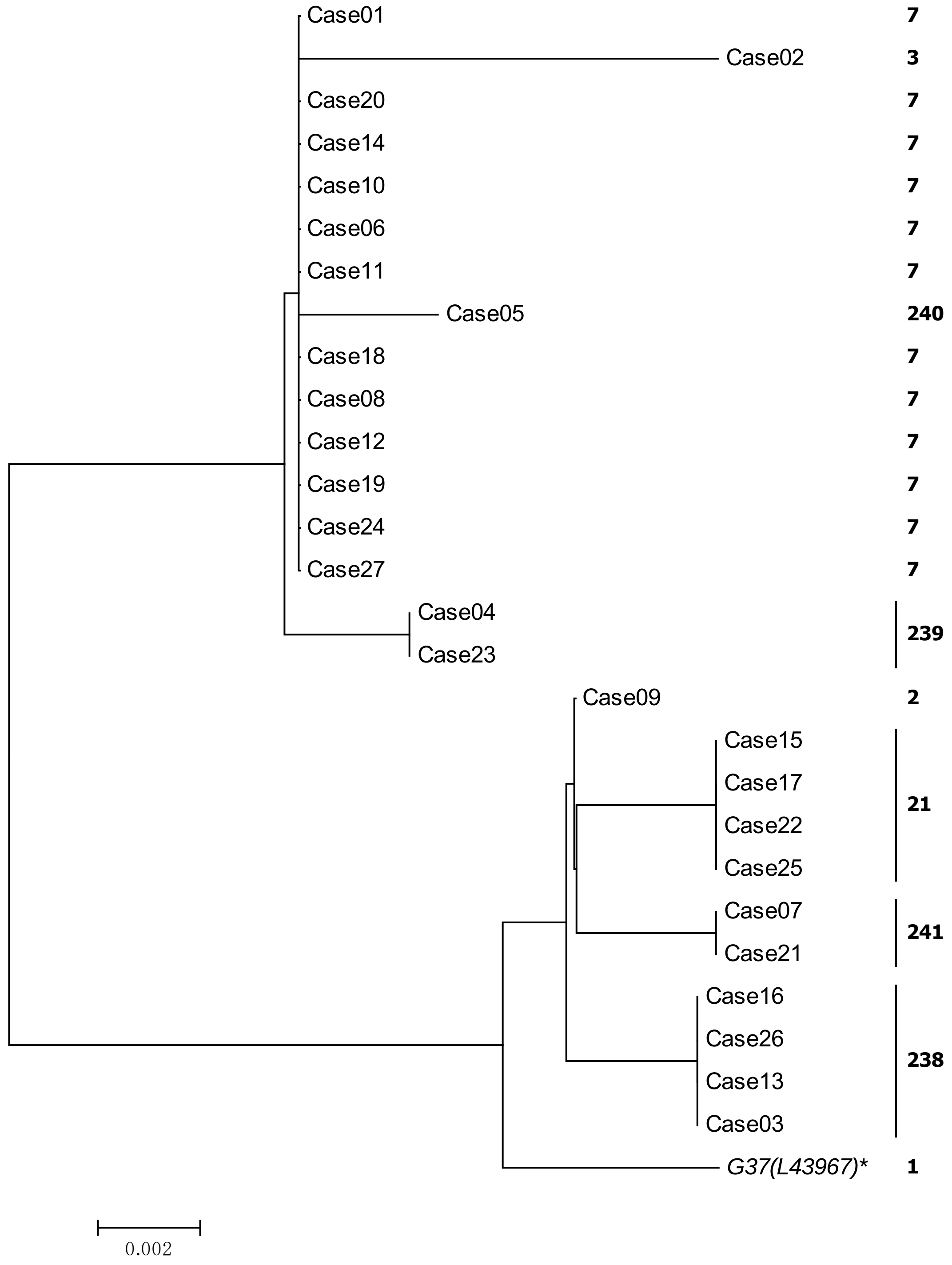

Supplementary Figure S2. Analysis of the number of tandem repeats in the MG309 locus of the lipoprotein gene in the 27 strains of *Mycoplasma genitalium* with A2058T mutations in the 23S rRNA gene detected in Gipuzkoa (2014-2021). The nucleotide alignment includes a small fragment of the amplified region (~350 bp), corresponding to the reference strain of *M. genitalium* G37 (GenBank accession number L43967*) between nucleotides 1,714 and 1,767 (the first and the last 9 nucleotides, in yellow). The region inside the square represents the triplet repeats (ACT, in green; ATT, in blue). The crosses (in grey) indicate the gaps introduced to optimise the alignment.

No. (%) patients	No. and distribution of repeats	AAAAGGATC	ACT	ACT	ACT	ACT	ATT	x x x	x x x	x x x	x x x	x x x	ACT	ATT	ACT	ATT	ACT	ATT	ACT	TGTATTTGA	*	MG37.genMG309-54pb
2 (7.4)	8	AAAAGGATC	ACT	xxx	x x x	x x x	x x x	x x x	x x x	x x x	x x x	x x x	ACT	ACT	ACT	ATT	ACT	ATT	ACT	TGTATTTGA		
15 (55.6)	9	AAAAGGATC	ACT	ACT	x x x	x x x	x x x	x x x	x x x	x x x	x x x	x x x	ACT	ACT	ACT	ATT	ACT	ATT	ACT	TGTATTTGA		
4 (14.8)	10	AAAAGGATC	ACT	ACT	ACT	x x x	x x x	x x x	x x x	x x x	x x x	x x x	ACT	ACT	ACT	ATT	ACT	ATT	ACT	TGTATTTGA		
6 (22.2)	11	AAAAGGATC	ACT	ACT	ACT	ACT	x x x	x x x	x x x	x x x	x x x	x x x	ACT	ACT	ACT	ATT	ACT	ATT	ACT	TGTATTTGA		

Supplementary Table S1. Designation of new *Mycoplasma genitalium* MG191 types (238-241) based on previously described types (reference 31) and FASTA sequences. All the strains were aligned from a common start to a common end, corresponding to nucleotides 221749 - 222029 of the *Mycoplasma genitalium* reference strain G37 (accession number L43967).

Type 1: G37 (L43967)
AGTTGATGAAACCTTAACCCCTTGGACTTGAAACAATAACAACTTCTCTTCACTAAAGA
TTACTGGAGAGAACCCAGGATCATTTGGATTAGTAAGAAGCCAAAATGACAACTTAAA
TATTTCAAGTGTTACAAAGAATTCTAGTGATGATAATCTCAAGTATCTCAATGCTGTTG
AGAAATACCTTGATGGTCAGCAAAACTTTGCAATCAGAAGGTATGATAACAACGGTAG
AGCTTTATATGATATTAACTTAGCAAAAATGGAAAACCCCTCAACGG

Type 238 (cases 3, 13, 16 and 26): GenBank accession number ON933572
AGTTGATGAAACCTTAACCCCTTGGACTTGAAACAATAACAACTTCTCTTCACTAAAGA
TTACTGGAGAGAACCCAGGATCATTTGGATTAGTAAGAAGCCAAAATGACAACTTAAA
TATTTCAAGTGTTACAAAGAATGTTAGTGATGATAATCTCAAGTATCTCAATGTTGTTG
AGAAATACCTTGATGGTCAGCAAAACTTTGCAATCAGAAGGTATGATAACAACGGTAG
AGCTTTATATGATATTAACTTAGCAAAAATGGAAAACCCCTCAACGG

Type 239 (cases 4 and 23): GenBank accession number ON933573
AGTTGATGAAACCTTAACCCCTTGGACTTGAAACAATAACAACTTCTCTTCACTAAAGA
TTACTGGAGAGAACCCAGGATCATTTGGACTAGTAAGAAGCCAAAATGAGAACTTAAA
CATCGCAAGTGTTACAAAGAATGGTAGTGATGATAATCTCAAGTATCTCAATGATGTT
GAGAAATACCTTGATGGTCAGCAAAACTTTGCAATCAGAAGGTATGATAACAACGGTA
GAGCTTTATATGATATTAACTTAGCAAAAATGGAAAACCCCTCAACGG

Type 240 (case 5): GenBank accession number ON933574
AGTTGATGAAACCTTAACCCCTTGGACTTGAAACAATAACAACTTCTCTTCACTAAAGA
TTACTGGAGAGAACCCAGGATCATTTGGACTAGTAAGAAGCCAAAATGAGAACTTAAA
CATCGCAAGTGTTACAAAGAATGGTAGTGATGATAATCTCGAGTATCTCAATGCTGTTG
AGAAATACCTTGATGGTCAGCAAAACTTTGCAATCAGAAGGTATGATAACAACGGTAG
AGCTTTATATGATATTAACTTAGCAAAAATGGAAAACCCCTCAACGG

Type 241 (cases 7 and 21): GenBank accession number ON933575
AGTTGATGAAACCTTAACCCCTTGGACTTGAAACAATAACAACTTCTCTTCACTAAAGA
TTACTGGAGAGAACCCAGGATCATTTGGATTAGTAAGAAGCCAAAATGACAACTTAAA
TATTTCAAGTGTTACAAAGAATGTTAGTGGTGATAATCTCAAGTATCTCAATGCTGTTG
AGAAATACCTTGATGGTCAGCAAAACTTTGCAATCAGAAGGTATGATAACAACGGTAG
AGCTTTATATGATATTAACTTAGCAAAAATGGAAAACCCCTCAACGG

Reference 31: Dumke R. Molecular Tools for Typing *Mycoplasma pneumoniae* and *Mycoplasma genitalium*. Front. Microbiol. 13:904494, doi: 10.3389/fmicb.2022.904494.
